# To Correspondents

**Published:** 1858-09

**Authors:** 


TO CORRESPONDENTS.—Communications have been received from the following gentlemen, and will be noticed in due time:—Drs. Hamilton, Dunglison, Klugé, Kerr, Sim, Starley, Packard, Spilman, Todd, McClellan, Lopez, Hachenberg, Fischer, and Wright.

